# P66Shc (Shc1) Zebrafish Mutant Line as a Platform for Testing Decreased Reactive Oxygen Species in Pathology

**DOI:** 10.3390/jcdd9110385

**Published:** 2022-11-09

**Authors:** Landon Haslem, Jennifer M. Hays, Xin A. Zhang, Franklin A. Hays

**Affiliations:** 1 Department of Biochemistry and Molecular Biology, University of Oklahoma Health Sciences Center, Oklahoma City, OK 73104, USA; 2 Stephenson Cancer Center, University of Oklahoma Health Sciences Center, Oklahoma City, OK 73104, USA; 3 Department of Physiology, University of Oklahoma Health Sciences Center, Oklahoma City, OK 73104, USA; 4 Harold Hamm Diabetes Center, University of Oklahoma Health Sciences Center, Oklahoma City, OK 73104, USA; 5 Department of Nutritional Sciences, University of Oklahoma Health Sciences Center, Oklahoma City, OK 73117, USA

**Keywords:** zebrafish, mitochondrial dysfunction, apoptosis, ischemia/reperfusion, myocardial infarction, p66Shc, ROS, ShcA

## Abstract

Reactive oxygen species (ROS) dysregulation exacerbates many pathologies but must remain within normal ranges to maintain cell function. Since ROS-mediated pathology and routine cell function are coupled, in vivo models evaluating low-ROS background effects on pathology are limited. Some models alter enzymatic antioxidant expression/activity, while others involve small molecule antioxidant administration. These models cause non-specific ROS neutralization, decreasing both beneficial and detrimental ROS. This is detrimental in cardiovascular pathology, despite the negative effects excessive ROS has on these pathologies. Thus, current trends in ROS-mediated pathology have shifted toward selective inhibition of ROS producers that are dysregulated during pathological insults, such as p66Shc. In this study, we evaluated a zebrafish heterozygote p66Shc hypomorphic mutant line as a low-ROS myocardial infarction (MI) pathology model that mimics mammalian MI. Our findings suggest this zebrafish line does not have an associated negative phenotype, but has decreased body mass and tissue ROS levels that confer protection against ROS-mediated pathology. Therefore, this line may provide a low-ROS background leading to new insights into disease.

## 1. Introduction

Although once considered a dangerous physiological byproduct, ROS are now classified as important cell signaling molecules that are essential to normal cell functions such as survival, growth, and apoptosis [1,2,3]. However, stringent ROS regulation prevents deleterious effects, since excessive ROS promotes apoptosis and ROS deficiency impairs regular ROS signaling [1]. Thus, cells have developed intricate redox systems to balance ROS generation and neutralization [4,5,6,7,8]. The primary source of ROS within cells is the electron transport chain, but other important ROS producers include NADPH oxidase (NOX), NOX homologues, myeloperoxidase, xanthine oxidase, monoamine oxidase, and p66Shc [2,9,10,11]. P66Shc is a superoxide anion radical producer that regulates outcomes for a variety of ROS-mediated pathologies by acting as a molecular rheostat, promoting survival in normal conditions and during transient stress spikes, but promoting apoptosis when p66Shc ROS production is dysregulated. p66Shc-mediated apoptosis occurs via mitochondrial H_2_O_2_ and superoxide anion accumulation, which results in cytochrome c release from mitochondria, caspase cascade activation, and cell destruction [12]. Dysregulated p66Shc ROS activity negatively contributes to many pathologies including cancer, wound healing, aging, endothelial dysfunction, neurodegenerative disease, chronic obstructive pulmonary disease, organ failure, sepsis, diabetes, and ischemia/reperfusion injuries (IRI) [13,14,15,16,17,18,19,20,21,22,23,24,25,26,27,28]. We recently published a comprehensive review of p66Shc function in cardiovascular pathology [29].

ROS can exist as radicals or in non-radical forms. Non-radical ROS are generally less reactive than radical ROS and can participate in inter-compartmental signaling, whereas radical ROS are more reactive and are therefore less able to travel between cellular compartments before reacting with another molecule [30,31]. Since p66Shc ROS dysregulation causes pro-apoptotic mitochondrial H_2_O_2_ and superoxide anion accumulation, removing this pro-apoptotic signal without affecting p66Shc’s membrane function as an adaptor protein can provide new insights into how decreased ROS can affect various pathologies. Removing p66Shc ROS overproduction may greatly affect pathological outcomes since p66Shc produces ~30% of intracellular ROS fluorescence signals and because p66Shc knockout cell lines and zebrafish hypomorphic mutant lines are characterized by decreased oxidative stress and resistance to ROS-mediated pathology, respectively [12,32]. Although ROS are an important determinant for many disease states, ROS manipulation using small molecules has been met with recurrent failure and sometimes with detrimental outcomes. These results are considered an effect of non-specific ROS removal that neutralizes pathological and physiological ROS [33,34,35,36,37,38]. Therefore, new treatments for ROS-mediated pathology now focus on targeting only ROS sources that are dysregulated during pathology, such as p66Shc [33,39]. For this reason, a new zebrafish p66Shc hypomorphic allele line was characterized in the ROS-mediated model of myocardial infarction (MI). This zebrafish line has no gross abnormalities but has decreased tissue ROS and is resistant to ROS-mediated MI effects. Therefore, this line may provide valuable insights into other conditions by evaluating different disease models on a low ROS background.

**Figure 1:**
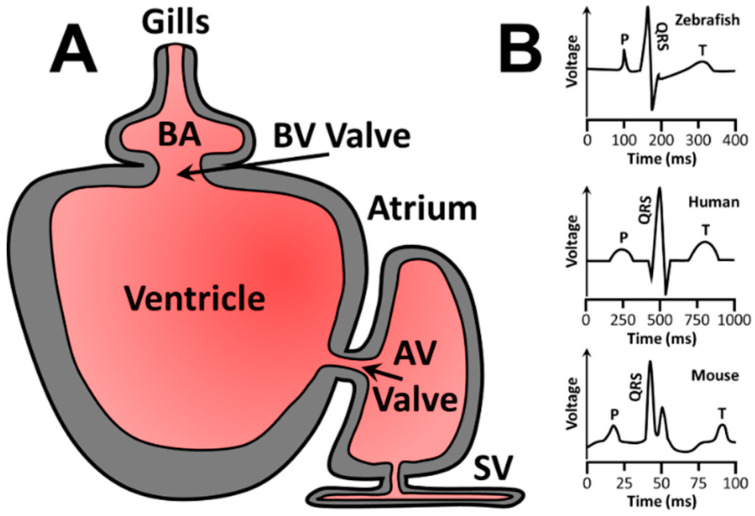
Zebrafish cardiac circulation diagram and representative ECG for zebrafish, humans, and mice. (**A**) Zebrafish cardiac circulation begins at the sinus venosus (SV), enters the atrium, passes through the atrioventricular (AV) valve, fills the ventricle, and is pushed through the bulbo-ventricular (BV) valve into the bulbus arteriosus (BA) before reaching the gills for oxygenation. (**B**) Representative ECGs of zebrafish (top), humans (middle), and mice (bottom). Human ECGs are more similar to zebrafish than mouse ECGs. P = p wave, or atrial depolarization. QRS = QRS complex or atrial repolarization combined with ventricle depolarization. T = T wave or ventricular repolarization.

The vertebrate *Danio rerio* (zebrafish) is a tropical freshwater fish, native to India. Zebrafish average 3.5–4.5 cm in length and have distinctive side stripes, from which their common name is derived. Zebrafish are used in many research models because most of their genes are shared with humans, with at least a single orthologous gene for 71.4% of protein-coding human genes [40]. Similarities between zebrafish and human cardiac electrophysiology support the utilization of zebrafish as an in vivo cardiac model. In this regard, zebrafish are more like humans than mice. For example, zebrafish have an average heart rate of 120–180 beats per minute, whereas humans and mice average 60–100 and 600 beats per minute, respectively [41,42]. In addition to similar heart rates, the zebrafish sinoatrial node analog is located in a similar manner to human sinoatrial nodes [43]. Zebrafish also demonstrate slow conduction, an electrophysiological property of heart conduction where electrical impulses are slowed between atria and ventricles to increase ventricle filling, output, and efficiency [44,45]. Electrocardiograms (ECG) are also more similar between humans and zebrafish than between humans and mice (Figure 1B) [46]. Zebrafish and human ECGs have definite *p*-waves (atrial depolarization), QRS complexes (atrial repolarization, ventricle depolarization), and T-waves (ventricle repolarization) with similar QT intervals (ventricular depolarization and repolarization) [44]. In addition, depolarization and repolarization in zebrafish occur via K^+^, Na^+^, and Ca^2+^ electrolyte movement across specific channels, like humans. These advantages have led to increasing zebrafish use for studying cardiovascular pathology and development of the zebrafish p66Shc hypomorphic mutant line featured below.

The cryoinjury model utilized in this study induces MI by applying a liquid N_2_-cooled probe to the exposed ventricle, causing localized necrosis and apoptosis [47,48]. This model induces trans-mural damage with a fibrotic response that is like mammalian MI and recapitulates MI without removing cardiac tissue [47,49]. Cryoinjury can be repaired by zebrafish, but it requires ~130 days and zebrafish exhibit decreased ventricle contractility after cryoinjury wound resolution, like human MI [47]. The longer time scale in cryoinjury models allows investigators to analyze different aspects and stages of MI depending on when hearts are harvested, as cardiac responses to cryoinjury have three stages: inflammatory, reparative, and regenerative [50]. The inflammatory stage lasts approximately three days from cryoinjury and is characterized by an innate immune response with leukocyte infiltration and inflammatory cytokine release that facilitates cellular debris removal and fibroblast recruitment [51]. This stage is essential to downstream repair, as an inhibited inflammatory response impairs injury site revascularization, cellular debris removal, and cardiomyocyte proliferation. These impairments lead to permanent scarring [52]. Platelets (thrombocytes in zebrafish) also regulate MI outcomes at this stage since they can improve outcomes by preventing hemorrhage but can also form aggregates that limit blood flow, negatively affecting ischemia and reperfusion [53]. As increasing H_2_O_2_ levels and thymosin β4 signaling cause progressive decreases in leukocyte infiltration and interferon-γ release, the inflammatory stage transitions to a reparative stage [54]. 

The reparative stage in zebrafish lasts 3–7 days post-MI and is mediated by cytokines such as TGFβ and activin, which promote collagen deposition by fibroblasts that have transitioned into myofibroblasts [51]. In addition, injury border tenascin C synthesis also regulates post-MI and cardiomyocyte infiltration in zebrafish. Like mammals, the zebrafish injury site forms a fibrin scar with a dense collagen core that plays an important role in wound repair because decreased scar deposition, caused by excessive TGFβ inhibition, prevents cardiac regeneration and can cause partial ventricle ruptures in zebrafish [51]. Thus, targeted inhibition of TGFβ or TGFβ activators during early dysfunction may improve MI outcomes. Of note, p66Shc activates TGFβ so its targeted inhibition may improve deleterious post-MI cardiac remodeling effects [12]. In addition to fibrin and collagen deposition in myocardium, fibronectin is deposited at the affected epicardium while cardiomyocytes near the epicardium express integrin αVβ3. Resultant integrin β3–fibronectin interactions are necessary for zebrafish wound resolution [55]. The final, regenerative, phase lasts 8–130 days post-cryoinjury and is characterized by a variety of cellular processes that ultimately result in cardiomyocyte proliferation, revascularization, and epithelial-to-mesenchymal transitions that involve endocardium, epicardium, and myocardium [50]. 

**Figure 2:**
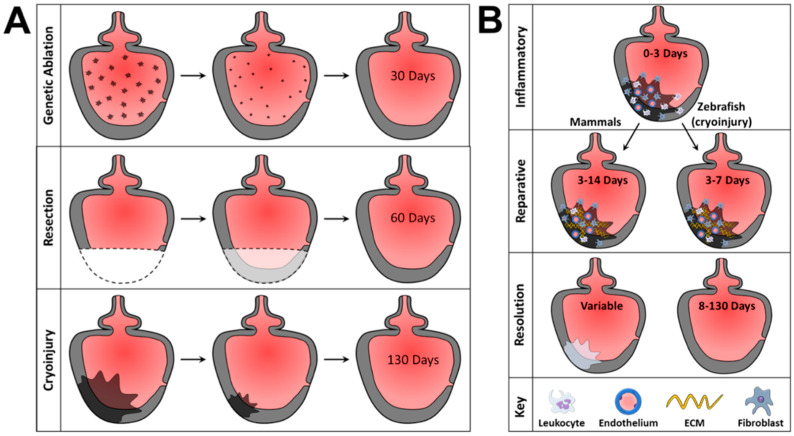
Damage patterns in MI models and MI wound repair process. (**A**) Representations of zebrafish MI models and their damage patterns. Genetic ablation targets only myocardium and is resolved in 30 days. Resection models are transmural but remove tissue that would otherwise require cellular debridement and cause extracellular matrix (ECM) protein deposition that is inconsistent with mammalian MI. Resection injury resolves in 60 days. Cryoinjury is a transmural injury model that closely resembles mammalian MI, requiring cellular debridement and showing ECM protein composition and deposition patterns like those observed in mammalian MI. (**B**) Stages of wound repair in mammalian and zebrafish cryoinjury MI by stage. The inflammatory stage lasts from 0 to 3 days of injury and is characterized by inflammatory cell recruitment and inflammatory responses governed by the innate immune system. The reparative phase lasts 3–14 days in mammals or 3–7 days in zebrafish cryoinjury models. During this stage, ECM deposition provides a scaffolding for wound resolution. Wound resolution ends with characteristic loss of cardiomyocytes, scarring, and decreased cardiac function in mammals, while zebrafish can regenerate lost cardiomyocytes from cryoinjury within 130 days of injury.

Since humans have limited cardiac regenerative capacity, zebrafish cardiac injury at the end of the reparative phase (before regeneration starts) is the most similar time point to initial post-MI human injury. The MI wound repair process is summarized in Figure 2B. This study harvested zebrafish hearts at a timepoint (~7 days) when mammalian and zebrafish MI pathologies were most similar and assesses a p66Shc hypomorphic mutant to evaluate low-ROS background and MI response. The results support this zebrafish line functioning as a low-ROS platform which does not introduce detrimental effects, and can be used to study ROS effects in other pathologies.

## 2. Materials and Methods

### 2.1. Zebrafish Line Creation

Zebrafish were obtained from the Zebrafish International Resource Center (ZIRC). ZIRC catalog ID ZL12658.11 hypomorphic allele female fish were outcrossed for three generations with myl7:eGFP (wildtype, WT) homozygotes before experiments or characterization began. Fish were housed in a temperature-controlled aquatic colony (28.5 °C, 14-h light and 10-h dark circadian cycle). Control fish were non-mutant clutchmates or myl7:eGFP homozygotes. Fish were genotyped via tail clip DNA extraction and PCR using the following sequences as primers: GTG AAT GTC GTT CTG GCT CG (reverse, IDT), TTG GCT TCC AAA GAG AGA CTT CA (forward, IDT). Fish were anesthetized with 0.02% tricaine methanesulfonate (MS-222) during tail clippings. Phusion^®^ High-Fidelity DNA Polymerase (New England Biolabs, Ipswich, MA, USA) was used for PCR sequencing. PCR reactions were cleaned using Monarch PCR & DNA cleanup kits and protocols (New England Biolabs, Ipswich, MA, USA). Amplicons were sequenced using the primers above (IDT, Newark, NJ, USA). Young healthy adult fish between 6–8 months age were used for cryoinjury. Male and female fish were randomly used. Animal experiments were conducted under the approved protocol (19-058-SAHB) from the OUHSC Institutional Animal Care and Use Committee, which conforms to the Guide for the Care and Use of Laboratory Animals, 8th Edition published by the National Institutes of Health.

### 2.2. Cryoinjury Procedure

Cryoinjury was performed as previously described [48]. Briefly, anesthetized fish (0.032% (*w*/*v*) MS-222) were immobilized in a sponge holder. A dissecting microscope was used to make a small incision that exposed the pericardial sacs that were partially removed with forceps. Then, applying gentle abdominal pressure caused the ventricles to protrude from the pericardial sacs. Hearts were dried with paper towels before applying a 0.3 mm diameter copper probe (precooled with liquid nitrogen for a minimum of one minute) to the ventricle for 30 s to induce MI. Mechanical damage was limited by avoiding probe movement during cryoinjury. After cryoinjury, fish were resuscitated using a pipette to move water across their gills until regular breathing was observed. Cryoprobes were cleaned between surgeries from fish in a single tank using tank water and paper towels. Between tanks, cryoprobes were cleaned with 70% (*v*/*v*) ethanol solutions. Sham surgeries followed the same procedure but the probe was not pre-cooled with liquid nitrogen for shams. The cryoprobe was constructed as suggested [48].

### 2.3. Organ Collection

Before organs were collected, fish were anesthetized (0.02% MS-222) and euthanized in ice water 7–8 days after cryoinjury. Incisions from initial cryoinjury were expanded before hearts were released by cutting between the atrium and sinus venosus. After hearts were removed, brain, skeletal muscle, liver, ovary, intestines, kidney, brainstem, and eyes were removed. Reported statistics are relative to cryoinjured WT fish. Potential confounding variables were limited by performing surgery and recovery under identical conditions and in the same location. At least three fish were used in each group and only fish that died during surgery or before tissue harvest were excluded from analysis.

### 2.4. Organ Preparation for Histology and Protein Quantification

Harvested organs were immediately placed in 10 mL of 10% (*v*/*v*) neutral buffered formalin (VWR, Radnor, PA, USA) and kept on ice until transferred to a 4 °C cold room, where organs were fixed overnight on a rocker. Organs were rinsed with 1× PBS, pH 7.4, for 10 min (3×) before embedding in paraffin or Tissue-Plus OCT (Thermo Fisher Scientific, Waltham, MA, USA). For histology, organs were cut in 6 µm serial sections using a CryoStar NX70 (Thermo Fisher Scientific, Waltham, MA, USA) and stained with MitoSox or H&E (Thermo Fisher Scientific, Waltham, MA, USA), or Trichrome (Polysciences, Warrington, PA, USA). Histological differences were quantified using ImageJ software. H&E and trichrome images were collected by an Olympus VS120 virtual slide system (Olympus Life Sciences, Waltham, MA, USA). MitoSox images were collected with a Eclipse Ts2 fluorescent microscope (Nikon, Melville, NY, USA). Mean technical replicates (3–6 per parameter) were used to represent effects on a single heart. A minimum of three hearts were used for all quantified parameters. Protein quantification experiments used corresponding organs from seven different wildtype male, wildtype female, p66Shc^−/+^ male, or p66She^−/+^ female fish for a total of N = 7 per quantification.

### 2.5. Histology and Protein Quantification

Superoxide staining was performed with 5 µM MitoSox, as previously described in fixed cardiac tissue [56]. MitoSox is a fluorescent dye that targets mitochondria and is selective for superoxide anion [57]. Staining was performed in the dark, at room temperature (RT), for 20 min. MitoSox staining was followed by 1-min washes (5×) in 1× PBS before mounting slides with ProLong™ Gold (Thermo Fisher Scientific, Waltham, MA, USA) antifade mountant containing DAPI counterstain. Mounted slides were covered from light and set overnight on a level surface, and imaged the following day. Fluorescence measurements were performed with NIS-Elements BR software (Nikon, Melville, NY, USA). H&E staining was performed using Harris’ protocol as previously described [58]. Trichrome staining was performed per the manufacturer’s instructions (Polysciences, Warrington, PA, USA). Slides were incubated in preheated Bouin’s fixative for one hour. Fixative was rinsed under running water (5 min) before staining with Weigert’s hematoxylin (10 min). Hematoxylin was removed using gentle running water (5 min) before staining samples with Biebrich Scarlet—acid fuchsin (5 min). Slides were rinsed in deionized water (three changes) before a 10-min incubation in phosphotungstic/phosphomolybdic acid. After incubating with phosphotungstic and phosphomolybdic acid, slides were rinsed in deionized water for three changes, then transferred to aniline blue for 5 min. Slides were rinsed in deionized water and then transferred to 1% (*v/v*) acetic acid (1 min). Slides were dehydrated and mounted as described for H&E staining. For protein quantification, methods were performed as previously described [59,60] with minor modifications. Tissue was Dounce homogenized in 1 mL ice cold lysis buffer (2M thiourea, 2% (*v/v*) DTT, 20 mM Tris-HCl pH 7.4RT, 1% (*w/v*) DDM, 100 mM NaCl, and 1X DHALT protease inhibitor). Clarified supernatant was collected following centrifugation at 12,000× *g* for 20 min at 4 °C. Total protein concentration was estimated using the Bradford method with 20 µg total protein electrophoresed per well and transferred to PVDF membrane for Western blotting. Blots were incubated with anti-p66Shc antibody (LS Bio, Seattle, WA, USA) followed by secondary antibody per manufacturer’s protocol. Subsequent total protein normalization for densitometry used Ponceau S staining and Image Lab software version 3.0.1 (Bio-Rad, Hercules, CA, USA). 

### 2.6. Microscopy

After resection, hearts were rinsed with 1× PBS and images were taken under a dissecting microscope for light microscopy images. For SEM microscopy, hearts were placed in 10 mL of ice-cooled 1× PBS (pH 7.4) fortified with 4% (*w/v*) EM-grade glutaraldehyde. Hearts were fixed in a 4 °C cold room overnight on a rocker. Excess fixative was removed by washing (3×) samples in 1× PBS for 10 min. Post-fixation was performed via immersion in 1 mL of 1% (*v/v*) OsO4 in PBS. Hearts were washed (3×) in deionized water before 25%, 50%, 70%, 85%, 95%, and 100% (3×) sequential alcohol dehydration (*v/v*). Dehydrated hearts were critical-point-dried before mounting on SEM sample holders. Once mounted, hearts were sputter-coated with 5 nm AuPd before visualizing on a Zeiss Neon dual-beam HR-scanning electron microscope. Image settings are indicated on each image. SEM images were analyzed with ImageJ software (1.8.0). Cell classifications were based on characteristic morphological differences corresponding to cell type. All analyses included 2–5 technical replicates per heart with a minimum of three hearts analyzed per parameter. Each biological replicate was represented by the averaged technical replicate value. 

### 2.7. Physical Activity and Weight Ratios

Fish movements were recorded in tanks for a minimum of 60 s before cryoinjury and again before organ collection. Fish were weighed after activity measurements before cryoinjury and heart resection. Physical activity is presented as mean distance travelled in 60 s. Weight is expressed as the mean ratio of pre-surgical to post-surgical weight. All groups had a minimum of three biological replicates. 

### 2.8. Quantification and Statistical Analysis

Statistical significance was calculated with Prism software v 9.4.1 (Graphpad, San Diego, CA, USA). Statistical tests were unpaired two-tailed t tests, assuming Gaussian distribution during tests. No inclusion/exclusion criteria were pre-established. Excluded data points were identified via ROUT as outliers within GraphPad Prism prior to exclusion. *p*-values < 0.05 were significant. *p* values < 0.05, 0.01, 0.001, 0.0001 are indicated by 1, 2, 3, or 4 asterisks, respectively. Values are presented as mean ± SD, as noted in figure legends.

## 3. Results

### 3.1. Quantification of p66Shc Expression Level in Wildtype and Hypomorphic Allele Zebrafish

**Figure 3:**
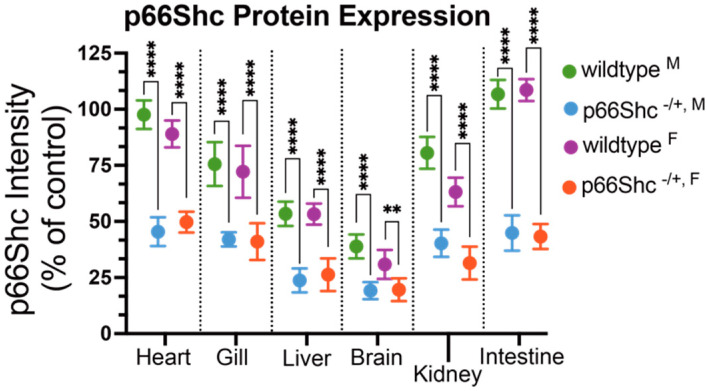
p66Shc protein expression in GFP^+/+^ (wildtype) and GFP^+/+^ p66Shc^−/+^ hypomorphic allele zebrafish. Heart, gill, liver, brain, kidney, and intestines were harvested from male (M) and female (F) zebrafish. Parental genotypes indicated in color (green and blue for male, purple and orange for female). **** = *p*-value < 0.0001, ** = *p*-value < 0.01, N = 7 independent fish for each measurement and presented as mean ± SD.

Hypomorphic allele zebrafish used in this study have an early obligate p66Shc splice site generating hetergozygous offspring (denoted as “p66Shc^−/+^”) when bred with wildtype fish. To assess resultant p66Shc expression level in various tissues, organs were resected from 13-month-old fish, homogenized, and ran on western blots (Figure 3). The only statistically significant (*p*-value < 0.0001) difference in p66Shc expression between male and female wildtype zebrafish was in the kidney (80.57 ± 7.14 in males vs. 63.14 ± 6.39 in females). This is consistent with prior data showing renal p66Shc expression was higher in mouse males and attributed to testosterone-dependent upregulation of p66Shc expression [61]. In addition, total p66Shc expression was variable across all tissues tested (for both male and female zebrafish, wildtype and the hypomorphic mutant) with brain and liver showing the lowest overall total expression levels. These data are consistent with prior Shc1 expression data in humans, where p66Shc is highly expressed in 23 out of 45 organs with liver, kidney, and brain showing attenuated expression relative to intestine, bone marrow, and pancreas [62]. Breeding p66Shc-deficient zebrafish with GFP^+/+^ wildtype zebrafish produces a statistically significant reduction in total p66Shc expression in all tissues tested (Figure 3). All reductions were significant (*p*-value < 0.0001), though female brain tissue produced a less significant reduction relative to other organs (*p*-value = 0.0083; 38.86 ± 5.34 in p66Shc^+/+, F^ vs. 30.86 ± 6.45 in p66Shc^−/+, F^). 

### 3.2. p66Shc Hypomorphic Allele Alters Embryo Diameter and Adult Zebrafish Mass

**Figure 4:**
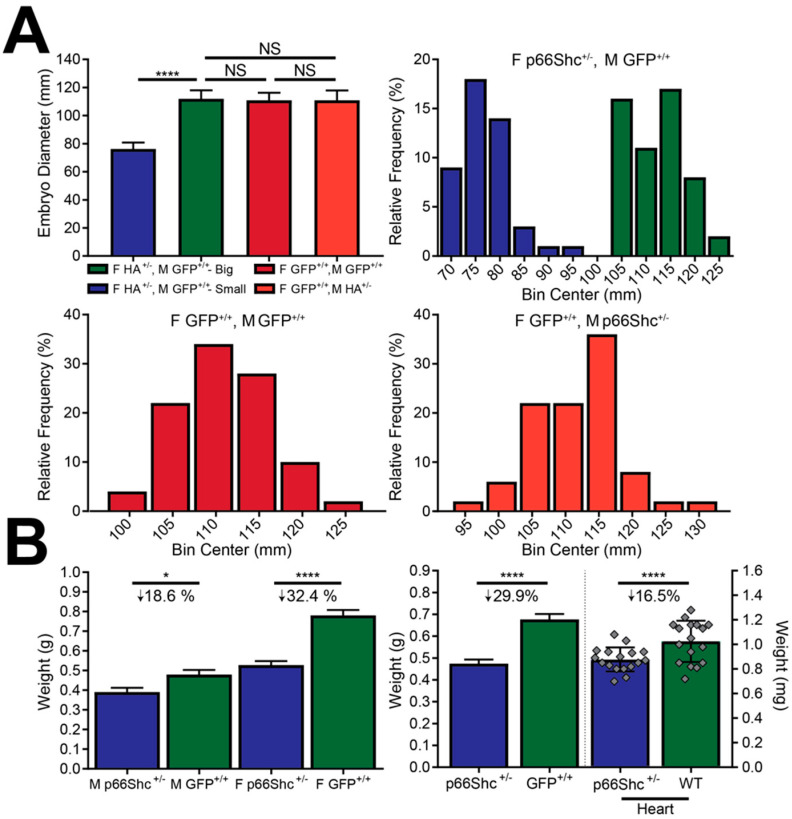
p66Shc hypomorphic allele embryo diameter and adult zebrafish mass. (**A**) Embryo diameter (top left panel) and embryo diameter frequency distribution of different parental crosses. p66Shc hypomorphic allele heterozygote females produce embryos with a bimodal size distribution that can be separated under a microscope. Size differences were significant and not observed when female WT (F GFP ^+/+^) fish were bred with male WT (M GFP ^+/+^) or male p66Shc^+/−^ (M hypomorphic allele (HA) ^+/−^). Parental genotypes are indicated by color and above their corresponding histograms. N = 50, **** = *p* < 0.0001, mean ± SD. (**B**) Whole fish weight differences (left panel) by gender and genotype. Males had a smaller mass decrease than females. Whole fish and heart mass differences (right panel), not gender stratified. N = 16 (hearts) or ≥ 36 (whole fish), * = *p* < 0.05, mean ± SD. Individual data points omitted for clarity.

When male WT fish were bred with female p66Shc heterozygotes, their 48-h-post-fertilization embryo sizes displayed a bimodal diameter distribution, but male heterozygotes bred with female WT fish did not. Upon visually separating small embryos from normal-sized embryos under a microscope, the smaller embryos showed a significant reduction in embryo diameter (Figure 4A). The smaller size was maintained through adulthood as p66Shc hypomorphic mutants had ~ 30% less body mass than their WT countertypes, N ≥ 36, *p*-value < 0.0001 (Figure 4B). Male p66Shc^−/+^ zebrafish had a smaller decrease in mass (18.6%) than females (32.4%). Organs also visually appeared smaller and showed decreased mass, with p66Shc^−/+^ hearts displaying a 16.5% mass reduction (Figure 4B, right panel).

Although breeding attempts produced homozygous p66Shc hypomorphic allele fish, no p66Shc^−/−^ fish were successfully raised. However, retroactive identification of a fungal infection suggests that the infection may have been responsible for the inability to raise p66Shc^−/−^ fish, since fish with unrelated genotypes also had difficulty breeding. This may also corroborate earlier findings where total p66Shc knockout is detrimental to mice in natural environments [63].

### 3.3. p66Shc Hypomorphic Allele Zebrafish Have Normal Organ Histology and Physical Activity but Decreased Tissue ROS Fluorescence

**Figure 5:**
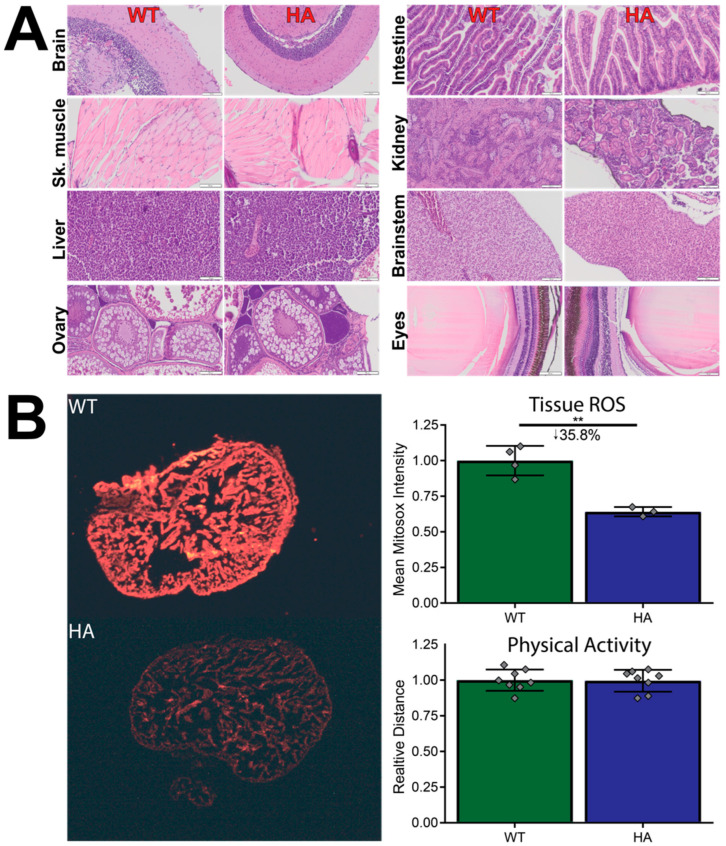
Tissue comparisons between WT and p66Shc hypomorphic allele zebrafish. (**A**) Representative H&E stains of WT and hypomorphic allele zebrafish organs. Organs do not show tissue-level abnormalities. (**B**) Physical activity is not affected in the p66Shc^−/+^ (HA) zebrafish line, but tissue ROS levels are significantly reduced. N = 8 for physical activity, 3–4 for tissue ROS. ** = *p* < 0.01, mean ± SD. Both images were uniformly contrast- and brightness-enhanced for clear visualization.

Further tissue analysis via hematoxylin and eosin (H&E) stains was performed on various zebrafish organs. Analysis indicates that, compared to WT controls, p66Shc^−/+^ fish had no gross morphological differences in the following organs: brain, skeletal muscle, liver, ovary, intestines, kidney, brainstem, and eyes (Figure 5A). Partial tissue separation in kidneys and eyes were artifacts introduced during slide preparation. Furthermore, pre-cryoinjury physical activity did not change between p66Shc^−/+^ and WT fish (Figure 5B). Yet, when hearts were analyzed for ROS content via MitoSox histological stains, the hypomorphic allele line showed a statistically significant 35.8% reduction in ROS fluorescence. Furthermore, fecundity and survival to adulthood did not appear to be affected by the p66Shc nonsense mutation. This suggests that, although smaller, hypomorphic allele fish maintain normal tissue function but with lower ROS concentrations. 

### 3.4. p66Shc Knockdown Zebrafish Have Improved Outcomes in ROS-Dependent Pathology

**Figure 6:**
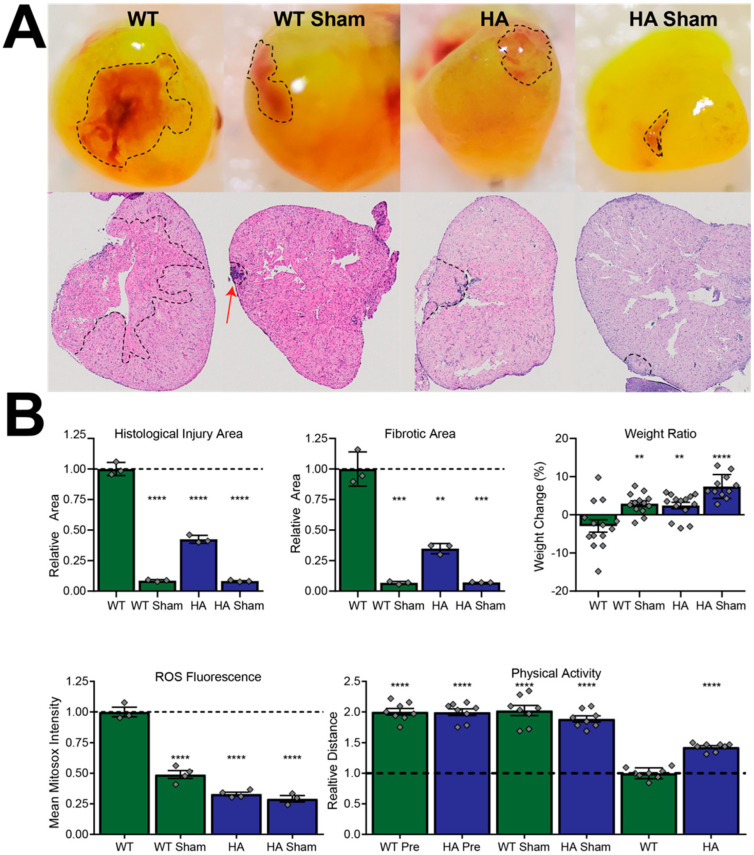
p66Shc hypomorphic allele improves MI outcome determinants in a ROS-dependent manner. (**A**) Representative stereomicroscopy images (top) and H&E stains (bottom) of resected hearts. (**B**) Quantification of zebrafish body mass effects, physical activity, and MI parameters from panel A. Hypomorphic allele zebrafish denoted as “HA”. **** = *p*-value < 0.0001, *** = *p*-value < 0.001, ** = *p*-value < 0.01, N = 7 independent fish for each measurement and presented as mean ± SD, N = 14 (weight ratio), N = 3 (injury and fibrotic area), N = 3 or 4 (ROS fluorescence), and N = 8 (physical activity).

To test the effects of ROS-dependent pathology on p66Shc^−/+^ zebrafish, fish were subjected to cryoinjury-induced MI. This model was chosen for a variety of benefits, including its close approximation to mammalian MI, as discussed above. Since MI severity is proportionate to MI ROS levels and because p66Shc produces ROS within mitochondria, partial p66Shc removal could provide a low-ROS platform that evaluates ROS-mediated effects in pathology [12,16,18,20,21,24]. In agreement with this hypothesis, p66Shc^−/+^ fish showed improvements in several parameters of MI pathology. Fluorescence microscopy revealed that p66Shc^−/+^ fish hearts, resected 1-week post-MI, showed a ~3-fold decrease in MitoSox ROS fluorescence signals, compared to WT fish with induced MI (Figure 6B).

The decrease in ROS fluorescence was associated with a visual difference in injury area. Histological quantification of injury area using H&E stains indicated that the decrease in ROS was associated with a ~2-fold reduction in damaged tissue area that showed similar decreases in trichrome fibrosis staining (Figure 6A). Furthermore, decreased ROS in hypomorphic allele lines was also associated with improvements in the organismal health indicators of post-MI physical activity (43.0%) and post-MI body mass (increased by 2.4% in hypomorphic allele fish, decreased by 2.9% in WT fish) (Figure 6B). 

**Figure 7:**
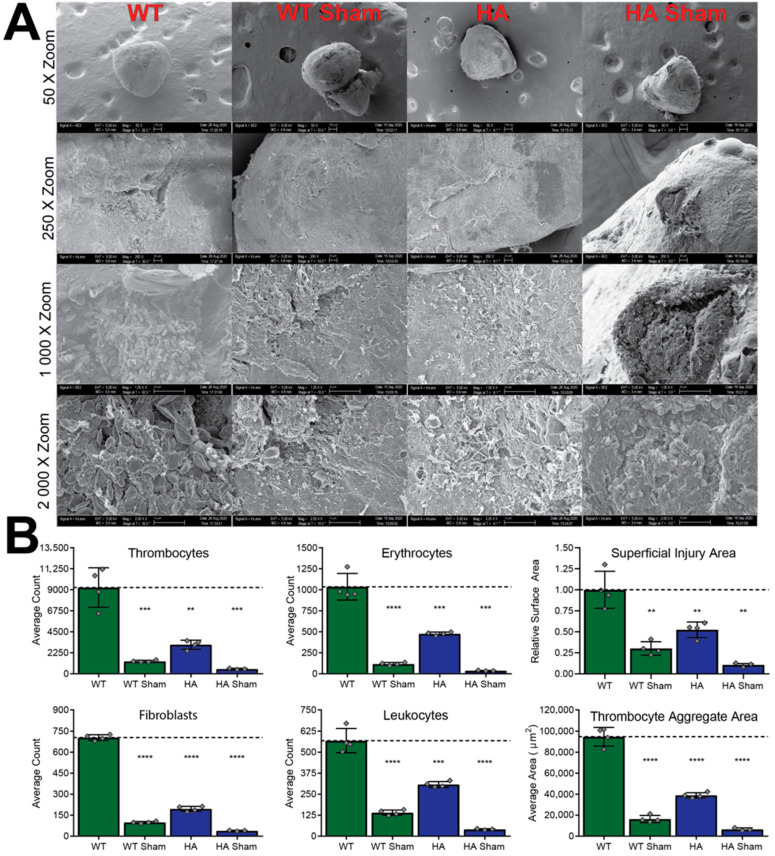
SEM cryoinjury imaging and associated quantifications. (**A**) Representative SEM imaging at four different zoom levels, illustrating differences between WT and hypomorphic allele (HA) groups. (**B**) Quantification of imaged parameters from panel A. **** = *p*-value < 0.0001, *** = *p*-value < 0.001, ** = *p*-value < 0.01.

In addition, scanning electron microscopy was used to quantify differences in sterile inflammation cell recruitment, thrombocyte aggregation, hemorrhage, and superficial injury area (Figure 7). Leukocytes, thrombocytes, and fibroblasts drive sterile inflammation and their recruitment was significantly decreased in the hypomorphic allele fish line by 45.8%, 66.2%, and 72.2%, respectively. Of note, hypomorphic allele fish were not more susceptible to infections than WT fish. Thus, decreased cell counts in the injured cardiac tissue are unlikely to indicate a loss in immune function. Thrombocyte aggregate areas and hemorrhage were also concomitantly decreased in hypomorphic allele fish, showing 58.9% and 53.9% respective decreases. Since hemorrhage and aggregate measurements show simultaneous decreases, the p66Shc hypomorphic allele does not appear to alter clotting activity. Lastly, hypomorphic allele fish had a 47.6% reduction in superficial injury area.

## 4. Discussion

In vivo ROS modulation models are limited, and the connection between ROS and normal function causes detrimental effects or phenotypes. This limits widespread applicability to research topics such as aging, electron transport chain functional analysis, or cancer [28,64,65,66]. Most in vivo models testing ROS effects produce non-specific ROS accumulation or neutralization via overexpression or silencing of antioxidant enzymes that can remove both beneficial and harmful ROS. However, p66Shc ROS overproduction is only associated with pathological states. Thus, partial p66Shc removal could mitigate the impact of deleterious apoptotic p66Shc ROS without adverse impact on normal cellular function. This is supported by research demonstrating that mitochondrial ROS levels determine MI outcomes through hormesis, with moderate superoxide anion levels conferring cardioprotection in mouse MI models [67]. 

Our results suggest that the generated p66Shc hypomorphic allele heterozygote zebrafish line mitigates ROS-mediated pathology, by limiting pathological p66Shc-mediated ROS output, without preventing regular ROS function. These results compliment additional findings from our lab focused on targeted inhibition of p66Shc function [12,29] using small molecules. Although these fish are smaller than their WT counterparts, there is no apparent pathological phenotype, nor observable histological alteration. The hypomorphic allele fish line did not have observable changes in fecundity, physical activity, or immune susceptibility during routine maintenance. Furthermore, since ROS fluorescence signals showed substantial ROS reduction in tissue, this fish line may represent a new in vivo genetic background for evaluating ROS effects on pathology with limited side effects. 

Underlying cell stress (e.g., derived from trauma, disease, or toxins) is a known mediator of increased intracellular ROS production leading to cytokine and growth factor production, fibrosis, and inflammation. This is demonstrated, in part, by the decreased number of activated fibroblasts in p66Shc-deficient (p66Shc^−/+^) zebrafish relative to wildtype fish (Figure 7B). Thus, the heterozygous p66Shc^−/+^ fish line could provide new insights into other ROS-mediated diseases (e.g., neurodegeneration, chronic obstructive pulmonary disease, peripheral wound healing, organ failure, sepsis, and stroke). Though current results are focused on p66Shc ROS-mediated diseases [13,14,15,16,17,18,19,20,21,22,23,24,25,26,27,28], the vision going forward is one of greater significance to general studies of ROS modulation disease phenotypes using this zebrafish system.

## Data Availability

Raw data presented in this study is available on request from the corresponding author.
